# Pd Single-Atom Sites on the Surface of PdAu Nanoparticles: A DFT-Based Topological Search for Suitable Compositions

**DOI:** 10.3390/nano11010122

**Published:** 2021-01-07

**Authors:** Mikhail Mamatkulov, Ilya V. Yudanov, Andrey V. Bukhtiyarov, Konstantin M. Neyman

**Affiliations:** 1 Boreskov Institute of Catalysis of the Siberian Branch of the Russian Academy of Sciences (BIC SB RAS), Novosibirsk 630090, Russia; mikhail@catalysis.ru (M.M.); avb@catalysis.ru (A.V.B.); 2 Departament de Ciència de Materials i Química Física and Institut de Quimica Teòrica i Computacional, Universitat de Barcelona, c/Martí i Franquès 1, 08028 Barcelona, Spain; 3 ICREA (Institució Catalana de Recerca i Estudis Avançats), Pg. Lluís Companys 23, 08010 Barcelona, Spain

**Keywords:** bimetallic nanoparticles, chemical ordering, density functional calculations, single-atom alloy catalysts

## Abstract

Structure of model bimetallic PdAu nanoparticles is analyzed aiming to find Pd:Au ratios optimal for existence of Pd1 single-atom surface sites inside outer Au atomic shell. The analysis is performed using density-functional theory (DFT) calculations and topological approach based on DFT-parameterized topological energy expression. The number of the surface Pd1 sites in the absence of adsorbates is calculated as a function of Pd concentration inside the particles. At low Pd contents none of the Pd atoms emerge on the surface in the lowest-energy chemical orderings. However, surface Pd1 sites become stable, when Pd content inside a Pd-Au particle reaches ca. 60%. Further Pd content increase up to almost pure Pd core is accompanied by increased concentration of surface Pd atoms, mostly as Pd1 sites, although larger Pd ensembles as dimers and linear trimers are formed as well. Analysis of the chemical orderings inside PdAu nanoparticles at different Pd contents revealed that enrichment of the subsurface shell by Pd with predominant occupation of its edge positions precedes emergence of Pd surface species.

## 1. Introduction

Metal nanoalloys constitute a modern class of materials important for many applications [1]. In catalysis, bimetallic nanosystems are often superior by their properties to the monometallic counterparts. For instance, there is a growing interest in a special class of bimetallic nanosystems, where a catalytically highly active metal, e.g., Pd or Pt, is distributed on the surface at sufficiently low concentration in the form of individual atoms embedded in the environment of a relatively inert metal, such as Au, Ag, or Cu [2,3,4,5,6,7]. These combinations allow solving a well-known problem whereby excellent reactivity of active metals often causes poor selectivity. Since the pioneering publications of Goodman and co-workers [2,3] PdAu nanoalloys are among the targets of active studies in the rapidly developing research field known now as *single-atom catalysis* [8,9], in particular in one of its branches dealing with *single-atom alloys* [10,11].

Gold-palladium system is a good example of a miscible alloy in the bulk. However, a lower surface energy of gold along with larger size of its atoms than palladium ones leads to surface enrichment by this element in thermodynamic equilibrium [12]. Under reaction conditions the surface thermodynamics may be alternated due to the stronger interaction of Pd with adsorbed species giving rise to surface segregation of Pd [13,14]. Since the chemical functionality of bimetallic nanoparticles (NPs) is intimately linked to their surface, a detailed understanding of nanoparticle architecture and control of its eventual changes under operating conditions are crucial for the rational design of nanosized materials with optimal catalytic performance [8,12].

Due to the complexity of bimetallic nanosystems the successful strategy to characterize their properties combines experimental and theoretical techniques [14,15]. At the computational level, density-functional theory (DFT) calculations provide quite reliable potential energies for metal nanosystems including bimetallic ones [16]. However, being efficient for nanosystems containing up to several hundred metal atoms, the DFT approach remains very computer-demanding for NPs comprising >10^3^ atoms. The scaling techniques based on DFT calculations of smaller NPs are available to reach larger size range for monometallic particles [17,18]. Besides the usual DFT modeling, ab initio molecular dynamics is a reasonable approach, although still quite expensive for routine applications to nanoalloy particles at sizes in the scalable regime. A promising approach for explicit simulations of bimetallic NPs employs simplified Hamiltonians, such as that of the embedded-atom method (EAM) parameterized on DFT data [19]. Another modeling approach relies on a DFT-parameterized topological energy expression (TOP method) [20,21] broadly applicable to bimetallic (*vide infra*) and other binary nanocrystals. The TOP expressions enable comprehensively exploring configurational space of locations (homotops) of two types metal atoms in different crystalline positions of bimetallic NPs with given nuclearity and morphology by Monte-Carlo (MC) minimization. Minimum-energy homotop in the explored configurational space provides thermodynamically stable distribution of two types of metal atoms over the lattice of the bimetallic NP under scrutiny, i.e., its equilibrium chemical ordering. Energies of other arbitrary chemical orderings can be also evaluated within the same approach [20].

Despite extensive studies of PdAu NPs by various theoretical approaches [19,20,21,22,23,24,25,26] there are still open questions on their equilibrium structure, even in the absence of adsorbates (under vacuum conditions). In particular, there is only a limited knowledge on the conditions for the presence of Pd on the surface, in terms of particle size and composition. If the content of Pd is small enough as, for instance, in Pd_0.25_Au_0.75_ composition, the outer shell totally consists of Au [19]. On the other hand, the compositions with a high Pd:Au ratio Pd_0.75_Au_0.25_ exhibit significant presence of Pd monomers, small ensembles, and islands even at low temperatures in the NPs with a wide size range, from ~100 to 5000 atoms [19]. For intermediate compositions, such as Pd_0.5_Au_0.5_, the situation is less clear: While for small NPs of ~100 atoms their interior is too small to accommodate all available Pd atoms, whose excess has to be distributed on the surface [21], for NPs larger than 1000 atoms the results provided by different approaches are slightly controversial, reporting either gold-coated NPs [21] or alloyed surfaces with substantial fraction of Pd species [19].

The present DFT and TOP study is performed with the goal to determine the conditions for appearance of single Pd atoms (monomers) on the surface of PdAu NPs. In contrast to the previous theoretical studies [19,21] based on the limited number of compositions, here a higher resolution with respect to Pd:Au ratio is explored, starting from a very low Pd fraction and gradually increasing it to about Pd_0.5_Au_0.5_. First, truncated-octahedral NP_201_ models with the fixed nuclearity and shape were addressed to study how distribution of the metal components within the NP—in the inner part and in the surface shell—changes with increasing Pd:Au ratio. The TOP technique was used to select the most stable configurations (homotops) applying DFT calculations to determine relative stability of these homotops. Further, the main findings were expanded to isomorphic NPs with higher nuclearity to find the Pd:Au ratio corresponding to the early appearance of Pd surface species on the particles with a given size. The ordered structures formed in the inner parts of these larger NPs at various Pd:Au ratios were also investigated.

## 2. Computational Details and Modeling Approach

DFT calculations were performed with the VASP code [27,28], employing PAW core potentials [29,30], and a gradient-corrected exchange-correlation functional by Perdew, Becke, and Ernzerhof (PBE) [31,32]. As in our previous work [14], for the determination of energetic descriptors from the topological expression (see Equation (1) below) a cut-off energy 250.9 eV was applied using the unit cell size 25 × 25 × 25 Å^3^ allowing a sufficient separation of 8 Å [33,34] between periodically repeated PdAu NPs composed of locally relaxed 201 atoms. As shown elsewhere [20,21], the usage of such somewhat less precise than common computational parameters in series of DFT calculations of metal NPs provides sufficient accuracy for determining the TOP energetic descriptors. In all calculations only Γ-point was used for the Brillouin zone sampling.

A modeling approach based on DFT calculations of highly symmetric particles [35,36,37] was applied to simulate the structure of bimetallic NPs. The main part of the present study is done with a truncated octahedral model NP_201_ at various Pd_x_Au_201−x_ compositions. This model, NP_201_, is small enough for extensive DFT calculations involving many Pd:Au configurations, each represented by a number of different topological arrangements (homotops) of Pd and Au atoms. On the other hand, such 1–2 nm large model NPs comprising few hundred atoms are already beyond the size range, where the quantum effects play role determining strong variation of properties [18]. Respectively, these model NPs belong to the scalable size range [18,35,38], which allows to extrapolate their properties to properties of NPs with 10^3^–10^4^ atoms corresponding to operational experimental model catalysts (3–7 nm) [13]. Thus, the DFT parameterization of the TOP method [20] obtained for the 201-atomic NPs Pd_x_Au_201−x_ is applied to study the distribution of Pd and Au atoms in the isomorphic 586- and 1289-atomic models Pd_x_Au_586−x_ and Pd_x_Au_1289−x_. This series of the truncated octahedrons is often referred to as Wulff particles, because the ratio of (111) and (100) facets in them obeys the Wulff construction conditions [14].

The topological energy expression defines the energy of each homotop as follows [20]:

(1) $$E_{TOP}=E_{0}+\varepsilon_{CORNER}^{Au}N_{CORNER}^{Au}+\varepsilon_{EDGE}^{Au}N_{EDGE}^{Au}+\varepsilon_{TERRACE}^{Au}N_{TERRACE}^{Au}+\varepsilon_{BOND}^{Au-Pd}N_{BOND}^{Au-Pd}$$

where $E_{0}$ is a constant offset between the TOP and DFT energy scales that cancels in the targeting homotops energy differences, $N_{BOND}^{Au-Pd}$ is the number of Au-Pd bonds (nearest-neighbor contacts) in the homotop; $N_{CORNER}^{Au}$, $N_{EDGE}^{Au}$, $N_{TERRACE}^{Au}$ are the numbers of Au atoms located in the corner, edge, and facet terrace positions on the surface of the homotop, respectively. The coefficients $\varepsilon_{Y}^{X}$ are energy descriptors representing energy contributions of either one Au-Pd bond or an Au atom located in the corresponding surface position of the NP to the total energy of the homotop, *E*_TOP_.

The DFT parameterization of Equation (1) for the Pd_56_Au_145_ composition of NP_201_ was obtained previously [14]. In the present work, the parameterization was performed for a number of other compositions of the NP_201_ model to account for the known dependence of the parameterization on the Pd:Au ratio [20]. The TOP energy descriptors obtained for the three compositions Pd_56_Au_145_, Pd_79_Au_122_, and Pd_101_Au_100_ with significantly different Pd:Au ratios are given in Table 1. The parameterization of Equation (1) for each of these models was made according to the previously developed protocol [20] by fitting to several dozen of DFT energies for distinct homotops representing a variety of Pd and Au atoms distributions with a wide range of energies. The descriptors for intermediate compositions between Pd_56_Au_145_ and Pd_79_Au_122_ ones were obtained using linear approximations based on the calculated descriptors for these two compositions (see Supplementary Materials, Figure S1, Table S1). For each Pd_x_Au_201−x_ composition under scrutiny the MC-minimization of Equation (1) carried out to select a set of 20–40 low-lying homotops within the energy interval up to 0.5–0.8 eV above the lowest state. Full structure optimization was performed to determine at the DFT level the (relative) energies of thus selected low-lying NP_201_ homotops (see energies and topological characteristics of studied homotops in Tables S2 and S3 of Supplementary Materials). In the chemical ordering calculations in the larger NP_586_ and NP_1289_ models the last verifying step by exceedingly demanding DFT calculations was omitted, fully relying on the *E*_TOP_ energies. Applications of this method [20,21] to various bimetallic NPs revealed that *E*_TOP_ expressions despite their simplicity are sufficient for very efficient determining reliable atomic distributions in the energetically most stable homotops, in good agreement with DFT data [14,15,39,40,41,42,43,44].

## 3. Results and Discussion

### 3.1. General Topological Analysis of Eventual Pd-Core/Au-Shell Segregation

The topological energy expression, Equation (1), due to its simplicity, defines a rather straightforward and clear physical model of Pd/Au ordering in the nanoalloy. The following general trends can be derived from this expression. The three terms with negative values $\varepsilon_{Y}^{Au}$ reflect the fact that Au is more stable on the surface, as a consequence of its lower surface energy and larger size of atoms. The stability increases in the order terrace < edge < corner with the decrease of the coordination of the corresponding sites. On the other hand, the negative $\varepsilon_{BOND}^{Au-Pd}$ descriptor is responsible for the known miscibility of PdAu alloy. Thus, the ordering in the inner part of NP reaches energy minimum at the maximal number of Pd-Au bonds, which is possible for a given Pd/Au ratio in the volume confined by the surface shell of the NP.

Evidently, to bring Pd to the surface the term $\varepsilon_{TERRACE}^{Au}$ should be compensated by a number of additional Pd-Au bonds formed via Pd-Au atoms permutation between the inner part and the surface of NP. If Pd fraction is so low that all Pd atoms can be distributed in the inner volume of NP as monomers, every Pd atom can form, at most, 12 Pd-Au bonds. Pd atoms located in the surface shell may form, at most, nine Pd-Au bonds on terraces and even less on edges and corners, seven and six, respectively. Hence, as follows from Equation (1), the core-surface Pd-Au permutations for such low Pd concentrations are endothermic:

$$\Delta E_{CORE↔TERRACE}=-\varepsilon_{TERRACE}^{Au}-3\varepsilon_{BOND}^{Au-Pd}$$

$$\Delta E_{CORE↔EDGE}=-\varepsilon_{EDGE}^{Au}-5\varepsilon_{BOND}^{Au-Pd}$$

$$\Delta E_{CORE↔CORNER}=-\varepsilon_{CORNER}^{Au}-6\varepsilon_{BOND}^{Au-Pd}$$

Applying the values of the corresponding descriptors obtained for the Pd_56_Au_145_ composition, the permutation energy equals 0.27, 0.47, and 0.53 eV for terrace, edge, and corner sites, respectively.

As an example of high Pd fraction, it is instructive to examine the stability of the ideal Pd-core/Au-shell structure in terms of Equation (1). In this case, the Au atom on the surface (111) terrace forms three bonds with Pd of the subsurface shell. Therefore, according to Equation (1), the energy associated with the (111) terrace position occupied by Au atom is $\varepsilon_{TERRACE}^{Au}+3\varepsilon_{BOND}^{Au-Pd}$. However, a permutation between Pd core and Au terrace creates additional Pd-Au bonds: Permuted Au atom in Pd inner core forms 12 Pd-Au bonds (if permutation involves deeper shells than the subsurface shell) and Pd atom in the surface shell forms six Pd-Au bonds with the neighboring Au atoms. Thus, the new homotop exhibits 15 more Pd-Au bonds than the ideal core/shell structure. Hence, the energy of such permutation $15\varepsilon_{BOND}^{Au-Pd}-\varepsilon_{TERRACE}^{Au}$ suggests that the permutated homotop is more stable than the core/shell one. The ratio $\varepsilon_{TERRACE}^{Au}/\varepsilon_{BOND}^{Au-Pd}$ for NP_201_ (Table 1) grows with increasing Pd content from 7.3 in Pd_56_Au_145_ to 10.1 and 12.8 in Pd_79_Au_122_ and Pd_101_Au_100_, respectively. Since the composition Pd_79_Au_122_ exhibits a homotop with the ideal core/shell structure (the core of NP_201_ consists of 79 atoms), the descriptors obtained for this composition seem to be most appropriate for estimating the energetics of core/shell structures. This example shows that the ideal core/shell structure is not the most stable for PdAu NPs and surface Pd monomers become stable before the core is completely filled by Pd. As shown in the following, the most stable Pd_79_Au_122_ homotops expose a considerable Pd fraction in the surface shell and surface Pd atoms become stable already at Pd:Au ratios notably lower than that in Pd_79_Au_122_.

Notably, increased Pd content may produce descriptors even with $\varepsilon_{TERRACE}^{Au}/\varepsilon_{BOND}^{Au-Pd}>15$, as in the previous studies [20,21], where smaller models than NP_201_ were used for the parameterization of Equation (1). For instance, the descriptors for Pd_70_Au_70_ model [20] yield $\varepsilon_{TERRACE}^{Au}/\varepsilon_{BOND}^{Au-Pd}=15.4$. In the present work the composition Pd_101_Au_100_ also exhibits the value 12.8 rather close to the critical threshold of 15, below which the homotops with ideal complete Au skins are predicted to become energetically competitive with homotops exposing isolated Pd atoms within such Au skins. At the compositions Pd_70_Au_70_ and Pd_101_Au_100_ the number of Pd atoms exceeds the size of the core of 44 atoms 1.6 times and of 79 atoms 1.3 times, respectively. Thus, application of such models for parameterizing Equation (1) would overestimate the stability of the core/shell segregation.

### 3.2. Pd Surface Concentration as a Function of Pd Fraction in the Core

Since the concentrations of two metal components on the surface and in the core (inside the outer atomic shell) of PdAu NP are different, we introduce the concentration/density of Pd atoms in the core as:

$$\rho_{core}^{Pd}=\frac{n_{core}^{Pd}}{n_{core}}$$

where $n_{core}^{Pd}$ is the number of Pd atoms located in the core and $n_{core}$ is the total number of metal atoms in the core. For NP_201_ $n_{core}$ equals 79 atoms (the outer shell consists of 122 atoms). The concentration (coverage) of Pd atoms on the (111) terraces is defined as:

$$\theta_{terrace}^{Pd}=\frac{n_{terrace}^{Pd}}{n_{terrace}}$$

where $n_{terrace}$ is the number of 9-coordinated atoms on the eight (111) facets, 56 for NP_201_, and $n_{terrace}^{Pd}$ is the number of Pd atoms in these terrace positions. Occupation of (001) facets by Pd starts rather late compared to (111) facets and just one Pd1 monomer is obtained in present work on (001) facet in the lowest-energy homotop of Pd_101_Au_100_ composition. Therefore, $\theta_{terrace}^{Pd}$ is defined here in terms of (111) terrace occupation.

**Figure 1:**
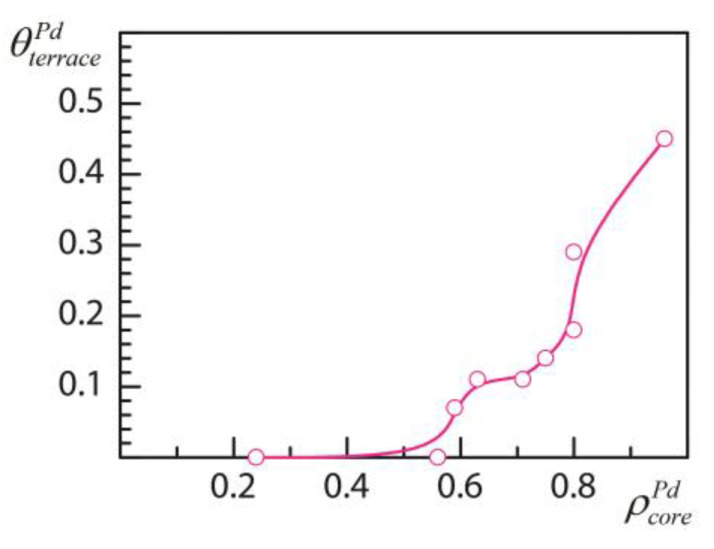
Concentration of Pd on the (111) terraces, $\theta_{terrace}^{Pd}$, as a function of Pd concentration in the core of NP_201_, $\rho_{core}^{Pd}$, calculated for different Pd_x_Au_201−x_ compositions (circles). See Table 2 for numerical data.

To find out how the distribution of two metal components within the NP framework changes with Pd:Au ratio it is instructive to consider the NP_201_ model at various Pd_x_Au_201−x_ compositions using DFT data of the low-lying homotops selected by MC minimization of Equation (1) (Table 2). Figure 1 shows $\theta_{terrace}^{Pd}$ as a function of $\rho_{core}^{Pd}$.

**Figure 2:**
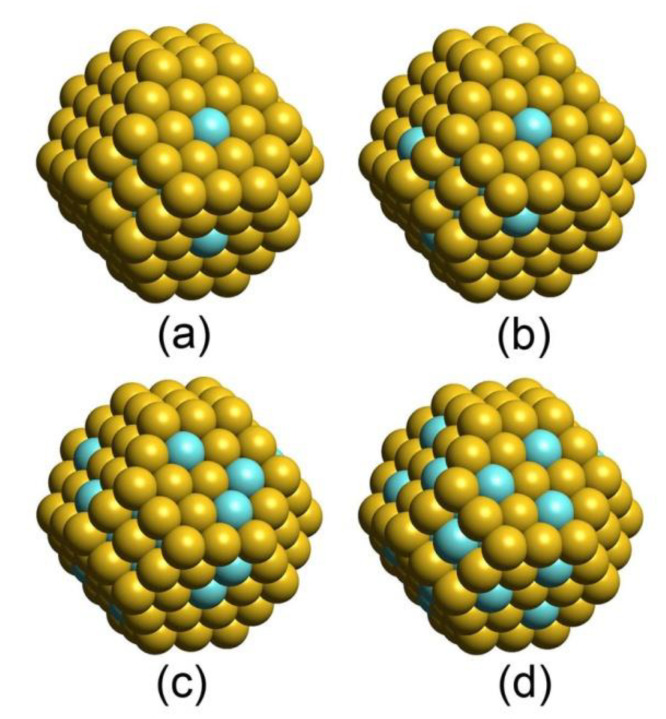
Views of the lowest-energy NP_201_ homotops with different elemental compositions: (**a**) Pd_51_Au_150_; (**b**) Pd_67_Au_134_; (**c**) Pd_79_Au_122_; (**d**) Pd_101_Au_100_. Pd atoms—light blue, Au atoms—yellow. Concentration of Pd on the surface grows with increasing Pd/Au ratio.

At low Pd:Au ratios there is no Pd at the surface (Table 2, Figure 1), and the structure of NP represents a bimetallic PdAu core surrounded by the one atomic layer thick purely Au shell. However, with the increase of Pd fraction, at $\rho_{core}^{Pd}$ about 0.6, Au-isolated Pd atoms (Pd1 monomers) appear on the surface, first at Pd_51_Au_150_ composition (Figure 2a). Further increase of Pd fraction leads to the more pronounced presence of Pd on the surface as shown in Figure 2 for the compositions Pd_67_Au_134,_ Pd_79_Au_122_, and Pd_101_Au_100._ At all considered compositions Pd is mostly distributed on the surface as Pd1 monomers. Pd2 dimers, when also present, are in much lower concentration. The surface location of Pd ensembles (monomers and dimers) is limited to (111) terraces. Only in the case of Pd_101_Au_100_, where the (111) terraces are almost saturated by Pd monomers (the saturation limit on NP_201_ is three Pd monomers per (111) facet that corresponds to $\theta_{terrace}^{Pd}$ = 0.43; further increase of $\theta_{terrace}^{Pd}$ should involve ensembles of two and more atoms), one Pd monomer appeared also on (001) facet. In addition, Pd_101_Au_100_ exhibits homotops, 0.3–0.4 eV above the lowest state, with single occupations of edge and corner sites by Pd. At none of other considered compositions was Pd found to be located in the lower-coordinated edge and corner sites, in line with the known higher stability of Au in low-coordinated positions of PdAu NPs [23] reflected by higher magnitudes of $\varepsilon_{CORNER}^{Au}$ and $\varepsilon_{EDGE}^{Au}$ descriptors than that of $\varepsilon_{TERRACE}^{Au}$ (Table 1).

Remarkably, the dependence of $\theta_{terrace}^{Pd}$ on $\rho_{core}^{Pd}$ shown in Figure 1 is essentially non-linear exhibiting two sharp increases, at $\rho_{core}^{Pd}$ ca. 0.6 and 0.8, and a rather flat plateau in between. Note, that a similar behavior was observed for PdAu (111) slabs using the Pd-Au EAM potential of Marchal et al. [19] in hybrid molecular-dynamics and MC simulations of Pd surface concentration as a function of Pd concentration inside a PdAu slab [45]. To rationalize the behavior shown in Figure 1 one has to consider the structural changes occurring inside the NP at growing $\rho_{core}^{Pd}$.

### 3.3. Ordered PdAu_3_ Structures Surrounded by Au Outer Shell

**Figure 3:**
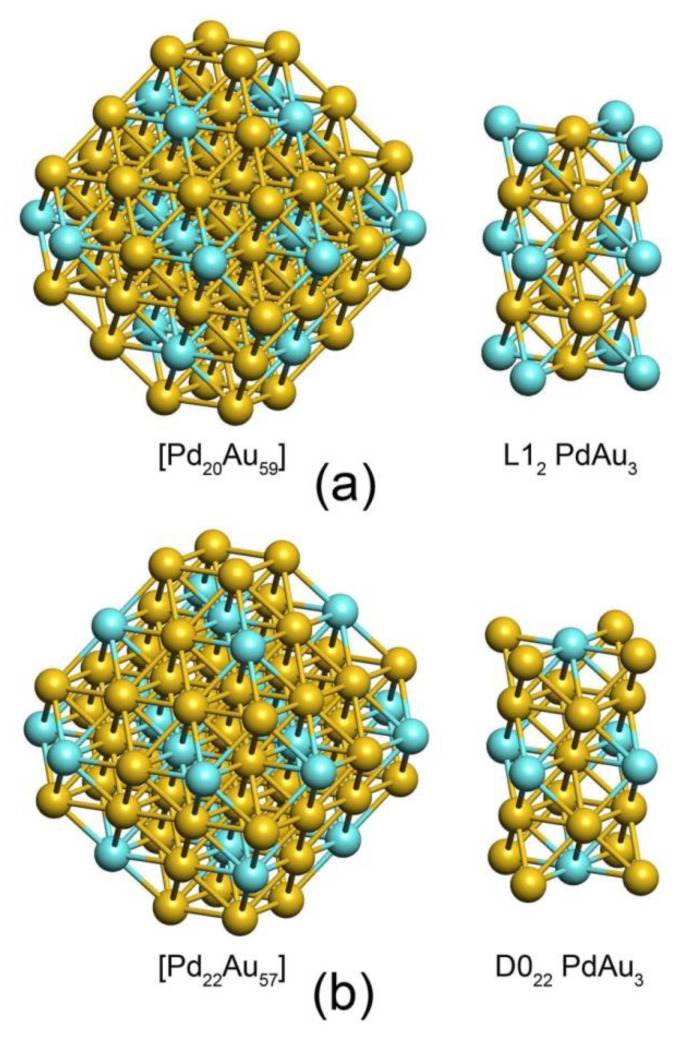
(**a**) The ordered core [Pd_20_Au_59_] (**left**) of Pd_20_Au_181_ NP with atomic order identical to the bulk phase L1_2_ PdAu_3_ (**right**). (**b**) The core [Pd_22_Au_57_] of Pd_22_Au_179_ NP with atomic order identical to D0_22_ PdAu_3_. All Pd atoms of the both structures are distributed inside the particles without contacting each other, i.e., there are no Pd-Pd bonds. Thus, the core structure represents PdAu alloy of approximately 1:3 ratio surrounded by a purely Au shell. The outer shell of Pd_20_Au_181_ and Pd_22_Au_179_ consisting of 122 Au atoms is not shown.

At low concentration Pd atoms tend to reside in inner part of the core of the model NP avoiding the direct contact to each other to maximize the number of energetically favorable Pd-Au contacts (the energy of two Pd-Au bonds is slightly larger than the energy of one Pd-Pd and one Au-Au bonds, as follows from comparison of homotops differing by a small number of bonds of different types). Thus, every Pd atom is coordinated by 12 Au nearest neighbors in all compositions up to Pd_22_Au_179_, which represents the limiting case with the maximal number of Pd monomers in the core of NP_201_. Remarkably, low-energy homotops of Pd_20_Au_181_ and Pd_22_Au_179_ compositions exhibit ordered structures identical to the structures of PdAu_3_ bulk ordered phases L1_2_ and D0_22_, respectively [46,47]. Figure 3 displays the frameworks of both NPs along with the corresponding bulk structures. The L1_2_ ordering was experimentally detected for ca. 5 nm large PdAu NPs [46]. According to DFT calculations of bulk alloys, both L1_2_ and D0_22_ structures exhibit rather high stability as manifested by the formation enthalpies −97 meV/atom and −101 meV/atom, respectively [47]. The realization of either L1_2_ or D0_22_ structures of such quite small particles as NP_201_ appears to be determined by Pd:Au ratio with atomic accuracy.

When the number of Pd atoms exceeds 22 (28% of total core atoms), Pd-Pd bonds start to be formed inside the core. For instance, there are two Pd-Pd bonds in Pd_23_Au_178_: a linear Pd3 trimer is formed via substitution of an Au atom coordinated by two Pd atoms in Pd_22_Au_179_ structure. The lowest-energy homotop Pd_24_Au_177_ already contains four Pd-Pd bonds: Two Pd2 dimers and one linear Pd3 trimer. Still, the main motif of the bulk-like ordered structure is preserved. Location of Pd atoms in the surface shell is unfavorable and the increase of Pd concentration/density in the core leads to increase of the total number of Pd-Au bonds, which stabilize the bimetallic system with respect to the monometallic counterparts.

### 3.4. Enrichment of Subsurface Shell by Pd Prior to Formation of Pd Surface Monomers

**Figure 4:**
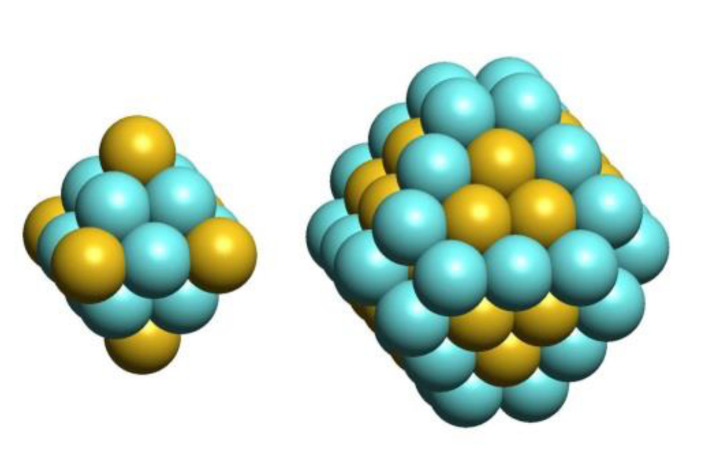
The ordered core structure [Pd_48_Au_31_] of Pd_51_Au_150_ and Pd_56_Au_145_ lowest-energy homotops. The first shell of 18 atoms (**left**) is built around the central Au atom to form a regular octahedron. The second shell (**right**) comprises 60 atoms. Completion of the outer shell (not shown) by 122 Au atoms leads to a stable Pd_48_Au_153_ homotop without surface Pd.

When the Pd fraction is increased up to about 60% of the NP core, the most stable homotops start to exhibit Pd1 monomers on the surface, see a sharp transition to non-zero $\theta_{terrace}^{Pd}$ in Figure 1. For instance, there are three and six Pd atoms on (111) facets (single Pd per facet) in the lowest-energy homotops of Pd_51_Au_150_ and Pd_56_Au_145_ compositions, respectively. The concentration of Pd in the core is 60 and 63%, respectively (Table 2, Figure 1). Here, the location of Pd atoms in the outer shell positions with the highest coordination number (9 for (111) facets) is in line with previous results [19,21] and directly follows from the topological energy expression. Structural analysis of the low-lying homotops Pd_51_Au_150_ and Pd_56_Au_145_ reveals that all of them are based on the ordered core [Pd_48_Au_31_]. The formula of the core is in brackets as a part of NP_201_ to distinguish it from an individual NP_79_ of the same nuclearity, which is often used in theoretical modeling. Inspection of the energetics of Pd_48_Au_153_ homotop shows that the structure with the ordered core [Pd_48_Au_31_] (see Figure 4) surrounded by the Au_122_ shell is stable to any Pd-Au permutation between the core and the outer shell. On the other hand, an additional Pd atom of the next composition, Pd_49_Au_152_, is stable on the surface. In terms of topological energy expression, Equation (1), the stabilization energy of a single Pd atom on the (111) facet of Pd_49_Au_152_ is

$$\Delta E_{CORE↔TERRACE}=-\varepsilon_{TERRACE}^{Au}+9\varepsilon_{BOND}^{Au-Pd}$$

Applying the descriptors optimized for Pd_56_Au_145_ composition one obtains exothermic effect $\Delta E_{CORE↔TERRACE}$ = −0.04 eV, which substantiates favorable location of additional Pd atom in the outer shell. The critical value to obtain a stable Pd atom in the surface shell for this configuration is $\varepsilon_{TERRACE}^{Au}/\varepsilon_{BOND}^{Au-Pd}<9$, that is the case for the topological descriptors obtained for compositions Pd_56_Au_145_ and Pd_67_Au_134_. However, the descriptors for compositions Pd_79_Au_122_ and Pd_101_Au_100_ with higher Pd content exhibit $\varepsilon_{TERRACE}^{Au}/\varepsilon_{BOND}^{Au-Pd}>9$. Therefore, they are not suitable for accurate enough representation of compositions with the lower Pd content.

Thus, the ordered core structure [Pd_48_Au_31_] is rather stable and at further increase of Pd fraction in the NP_201_ the excessive Pd atoms (>48) go to the surface forming “single atoms” on (111) facets. This structure [Pd_48_Au_31_] is also remarkable in the sense that this non-bulk-like ordering originates from the structural peculiarities of the NP. Pd atoms in the subsurface shell occupy edge and corner sites of the truncated octahedron [Pd_48_Au_31_], while Au atoms are located on (111) facets of the latter. Thus, the preference for Pd/Au ordering in the subsurface shell exhibits a reverse pattern to the outer shell ordering, where Pd first emerges on the facets leaving corner and edge sites to be more favorably occupied by Au atoms. Evidently, such distribution of Pd and Au atoms alternating with each other in the neighboring shells maximizes the number of Pd-Au bonds, thus increasing the stability.

### 3.5. Ordered PdAu Bulk-Like Structure

**Figure 5:**
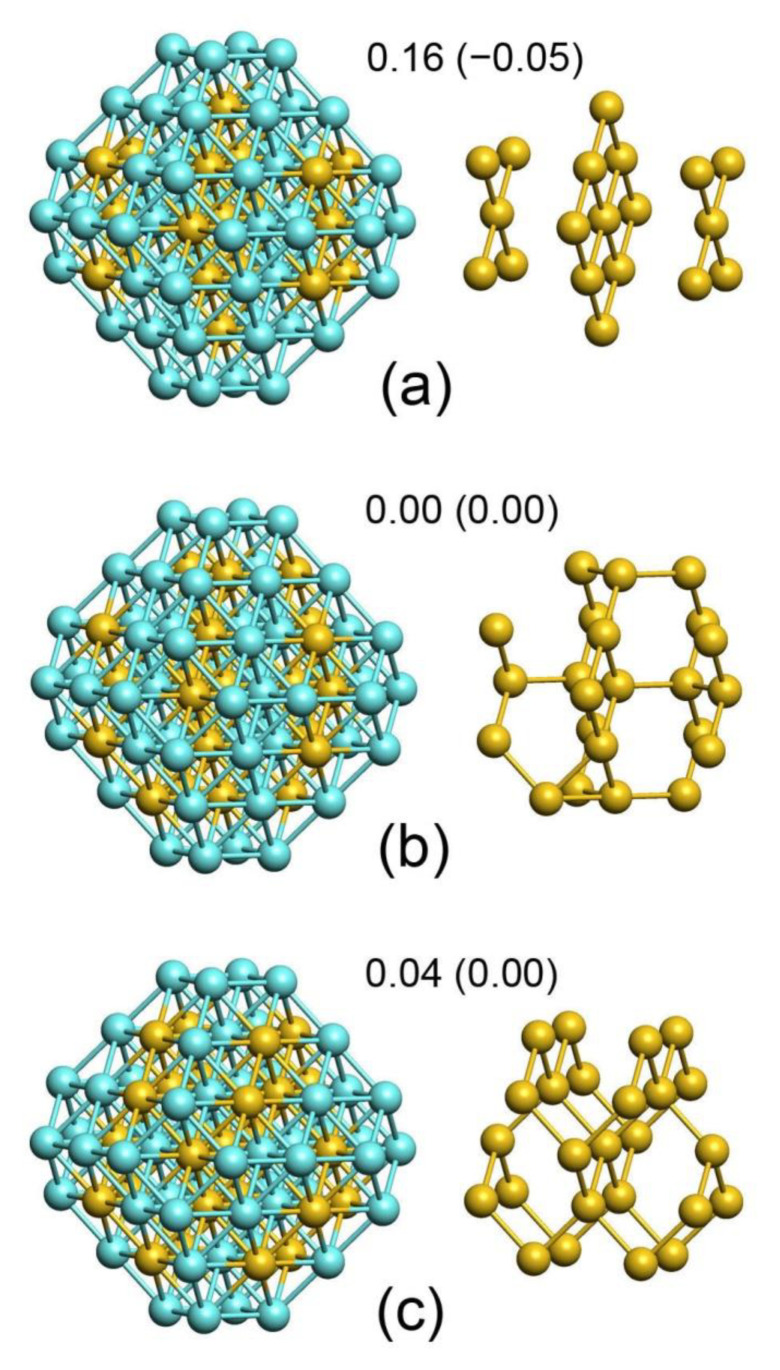
Core structures of Pd_62_Au_139_ homotops with different number of surface Pd atoms: (**a**) [Pd_60_Au_19_], (**b**) [Pd_56_Au_23_] and (**c**) [Pd_54_Au_25_]. The left column presents the complete core structures while the right column shows only Au atoms of the core for a clear view of the atomic ordering. The outer shells of the homotops (not shown) contain two (**a**), six (**b**), and eight Pd1 monomers (**c**). Relative energies of the homotops (in eV) obtained by DFT and TOP (in parenthesis) approaches are also presented.

Further increase of Pd fraction leads to a considerable increase of $\rho_{core}^{Pd}$ from 0.63 in Pd_56_Au_145_ to 0.71 and 0.76 in Pd_62_Au_139_ and Pd_67_Au_134_, respectively (Table 2). However, the number of six surface Pd atoms in the lowest-energy homotop Pd_62_Au_139_ remains the same as in Pd_56_Au_145_ and the number only slightly increases to seven in Pd_67_Au_134_. This results in a plateau in Figure 2 corresponding to $\theta_{terrace}^{Pd}$ ≈ 0.11. A detailed analysis shows that the composition Pd_62_Au_139_ exhibits several low-lying homotops with different numbers of Pd atoms in the outer shell and quite different patterns of the Pd-Au ordering in the inner core. Structures of three selected homotops are shown in Figure 5: [Pd_60_Au_19_]Pd_2_Au_120_ (5a), [Pd_56_Au_23_]Pd_6_Au_116_ (5b), and [Pd_54_Au_25_] Pd_8_Au_114_ (5c) with outer shells containing two (5a), six (5b), and eight (5c) Pd1 monomers. The TOP precision δ is limited for the studied 201-atomic PdAu NPs to 0.2–0.3 eV (Table 1) with the chosen structure of the topological expression (Equation (1)). This justifies the overestimated TOP stability of structures with low $\theta_{terrace}^{Pd}$ yielding the lowest energy for 5a with the ordered core [Pd_60_Au_19_] (Figure 5), while DFT gives a clear preference to higher $\theta_{terrace}^{Pd}$, as 5b and 5c, putting these homotops, respectively, 0.16 and 0.12 eV lower than 5a. At the TOP level, the energy difference between 5a,b is

$$\Delta E_{5a\to 5b}=-4\varepsilon_{TERRACE}^{Au}+30\varepsilon_{BOND}^{Au-Pd}$$

Thus, the homotop 5a has the lowest *E*_TOP_ with $\varepsilon_{TERRACE}^{Au}/\varepsilon_{BOND}^{Au-Pd}>7.5$ that is the case already for Pd_67_Au_134_ as well as for higher Pd:Au ratios (Table 1). Nevertheless, the DFT data favor the states with higher $\theta_{terrace}^{Pd}$, such as 5b,c with disordered core structures (Figure 5). We rely on DFT energies to make the final conclusions on Pd/Au distribution in NP_201_. However, the observations on TOP descriptors made in this work are important for better understanding the abilities and limitations of the TOP approach, especially when it is applied to large NPs, exceedingly demanding DFT calculations of which still remain impractical.

The homotops 5b,c are iso-energetic at $\varepsilon_{TERRACE}^{Au}/\varepsilon_{BOND}^{Au-Pd}=8$, since the energy difference between them is $\Delta E_{5b\to 5c}=-2\varepsilon_{TERRACE}^{Au}+16\varepsilon_{BOND}^{Au-Pd}$. In the absence of the explicitly calculated TOP descriptors for the composition Pd_62_Au_139_, one can estimate the value $\varepsilon_{TERRACE}^{Au}/\varepsilon_{BOND}^{Au-Pd}\approx 7.9$ by the interpolation of the descriptors listed in Table 1 for other compositions (see Supplementary Materials, Figure S1, Table S1 for more detail). On the other hand, 5a is the lowest-energy homotop according to DFT calculations with the energy just 0.04 lower than that of 5b homotop. This energy difference is too small to be conclusive. Nevertheless, it illustrates how sensitive is the value of $\theta_{terrace}^{Pd}$ to the values of TOP descriptors. A number of found homotops with significantly higher Pd surface fractions of 11–18 atoms have 0.2–1.0 eV higher energies than the lowest-lying homotops both at the DFT and TOP levels (see Supplementary Materials, Table S2). Applying the Boltzmann factor to define the populations of the lowest five homotops of Pd_62_Au_139_ (including 5b,c) at 300 K one gets the average $\theta_{terrace}^{Pd}$ = 0.126, which is only slightly higher than 0.107 corresponding to 5b homotop at 0 K.

The ordered core structure [Pd_60_Au_19_] of 5a is very peculiar because of its high stability along with high Pd density. If surrounded by purely gold shell, [Pd_60_Au_19_]Au_122_, this structure is stable to single permutations of Pd and Au atoms between the core and the skin. The arrangement of Au atoms in [Pd_60_Au_19_] (Figure 5a) is identical to L1_0_ PdAu bulk structure with Pd and Au layers alternating in [001] direction. The L1_0_ was observed experimentally for PdAu NPs [46]. According to DFT bulk calculations, L1_0_ phase is highly stable with the formation enthalpy −104 meV/atom; only PdAu bulk phase Nr 40 is slightly more stable with the formation enthalpy −118 meV/atom [47]. It is a remarkable feature of the nanostructure [Pd_60_Au_19_] that most of Au core atoms are located in the inner part of the core, which has the shape of a regular 19-atomic octahedron, with only four Au atoms in the subsurface shell. High Pd content in the subsurface shell compared to the inner core was reported on the basis of DFT-parameterized EAM modeling [19]. [Pd_60_Au_19_] core represents the situation of the subsurface shell almost completely, by 93%, composed of Pd.

Although in Pd_62_Au_139_ composition disordered cores with lower $\rho_{core}^{Pd}$ (5b,c) are preferred at the DFT level, the lowest-energy two Pd_67_Au_134_ homotops (with degenerate DFT energies) are based on the ordered core structure [Pd_60_Au_19_] (5a) with its high $\rho_{core}^{Pd}$, 0.76. Note, that another low-lying homotop (not further specified in the article) featuring only 0.01 eV higher energy exhibits the core [Pd_61_Au_18_] with even higher $\rho_{core}^{Pd}$ and contains elements of the bulk PdAu phase Nr. 40, the most stable for PdAu alloys according to DFT calculations [47]. Thus, Pd_67_Au_134_ composition is at the end of the flat part of the curve in Figure 1. Here, a rather low $\theta_{terrace}^{Pd}$ = 0.125 is accompanied by the subsurface shell composed of Pd by more than 90% and the atomic ordering in the inner core similar to that of PdAu bulk (with 1:1 ratio).

**Figure 6:**
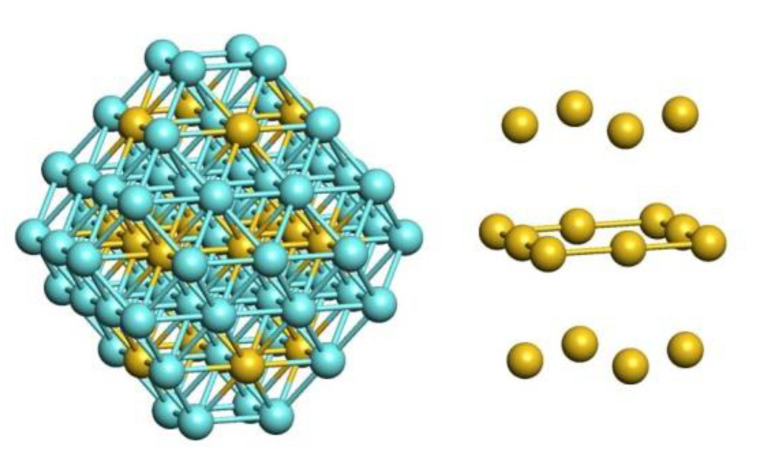
The ordered core structure [Pd_63_Au_16_] (**left**) and only Au atoms there (**right**) of the lowest-energy homotops Pd_73_Au_128_ and Pd_79_Au_122_.

Further increase of Pd fraction in NP_201_ leads to compositions Pd_73_Au_128_ and Pd_79_Au_122_ (Table 1), both of which exhibit in their lowest homotops the ordered core [Pd_63_Au_16_] (see Figure 6) with $\rho_{core}^{Pd}$ = 0.8 being only slightly higher than that of Pd_67_Au_134_ composition considered above. Similar to Pd_48_Au_153_ and Pd_51_Au_150_, where the transition to non-zero $\theta_{terrace}^{Pd}$ occurs, formation of a stable ordered core leads here to fast increase of $\theta_{terrace}^{Pd}$ (Figure 1) from 0.18 in Pd_73_Au_128_ to 0.29 in Pd_79_Au_122_ (Table 2). As at lower Pd content, the surface Pd fraction of Pd_79_Au_122_ is represented by Pd1 monomers surrounded by Au atoms on (111) terraces. Noteworthily, Pd_79_Au_122_ homotop with pure Pd-core/Au-shell structure is ca. 0.8 eV higher in energy (both at DFT and TOP levels) than the lowest-lying homotop.

Saturation of the NP core by Pd is reached in Pd_101_Au_100_ composition (Figure 1, Table 2). This configuration marks the following limits for occupation of core and terraces by Pd: (i) The core of NP in the lowest-energy homotops is completely (100%) or almost completely (96–98%) formed of Pd atoms; (ii) occupation of (111) terraces by Pd1 monomers in the lowest-energy homotops reaches 40% (22–24 Pd atoms). Three Pd atoms per terrace is the saturation limit for monomers on (111) terraces of NP_201_ (Figure 2d). Formation of Pd_n_ (n > 1) moieties on (111) facets and occupation of (001) facets by Pd start at higher Pd surface concentrations.

To summarize, the following picture emerges from the results obtained for NP_201_: (i) At low content Pd is completely located inside the core; (ii) when Pd concentration in the core reaches ca. 60%, Pd appears also on the surface in the form of single Pd1 centers (monomers) surrounded by Au atoms; (iii) at further increase of Pd:Au ratio the core becomes completely formed of Pd, and simultaneously the concentration of Pd1 species on (111) terraces reaches the saturation limit of ca. 40%.

### 3.6. Generalization to Larger Particles: NP_586_ and NP_1289_

PdAu NPs of model catalysts studied experimentally are 4–12 nm large with the mean size about 7 nm [13,14]. The theoretical model NP_201_ has a diameter *D* = 1.7 nm (the diameter of a sphere built on the vertex atoms of the truncated octahedron is meant here). To approach the size range of particles dealt with experimentally, the TOP method was applied to PdAu model particles NP_586_ and NP_1289_ with the same shape as NP_201_ and *D* = 2.6 and 3.5 nm, respectively. These particles are fully isomorphic with NP_201_ discussed in detail above. Their main differences from the smaller model NP_201_ are smaller surface/core ratios, which decrease with increasing particle size. The number of surface atoms in NP_201_ is 1.54 times larger than the number of core atoms (122 atoms on the surface and 79 atoms in the core). The notably smaller surface/core ratios, 0.87 for NP_586_ and 0.60 for NP_1289_, reflect that there are more atoms in the core than on the surface of these larger particles.

**Figure 7:**
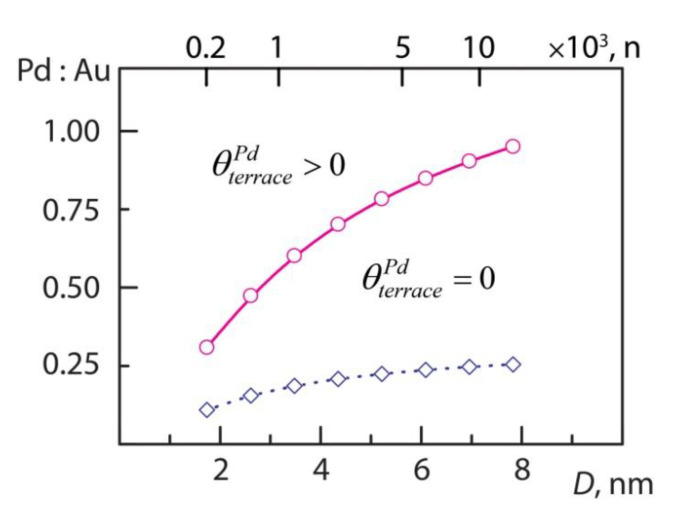
Conditions for existence of Pd surface species on the surface of PdAu truncated-octahedral NPs with a given Pd:Au ratio and diameter *D*, nm (the upper scale shows also the NP nuclearity, *n*). The solid line (circles) corresponds to Pd concentration $\rho_{core}^{Pd}=0.6$ in the core covered with Au shell ($\theta_{terrace}^{Pd}=0$). With the increase of $\rho_{core}^{Pd}$ above this line, Pd1 monomers and ensembles become stable on the surface of NPs ($\theta_{terrace}^{Pd}>0$); below is the region with Pd fraction residing inside the core surrounded by the purely Au shell. The dashed line (diamonds) corresponds to $\rho_{core}^{Pd}=0.25$, where a bulk-like PdAu_3_ ordering is expected. Below this line, $0<\rho_{core}^{Pd}<0.25$, Pd fraction is distributed in the form of single Pd atoms in the core of NPs.

A correspondence between the concentrations of metal components in the core and in the surface shell of PdAu NP_201_ (Figure 1) has been established in Section 3.2. To apply this correspondence to NPs of arbitrary size one has to take into account that at the same Pd:Au ratio the distribution of two metal components between core and surface may be size-dependent due to a varying surface/core ratio. For instance, in NP_201_ with 1:1 Pd:Au ratio (composition Pd_101_Au_100_), the number of Pd atoms exceeds the total number of the core atoms resulting in the core almost saturated by Pd ($\rho_{core}^{Pd}$ = 0.98). However, in NP_1289_ with the same 1:1 Pd:Au ratio (composition Pd_645_Au_644_), the number of Pd atoms is about 20% smaller than the number of the core atoms, 807. Therefore, according to Figure 1 considerable Au fraction in the core is expected in contrast to Pd_101_Au_100_. Furthermore, a notably lower $\theta_{terrace}^{Pd}$ is expected for Pd_645_Au_644_ compared to Pd_101_Au_100_. Since $\rho_{core}^{Pd}$~0.6 is shown to be the threshold value, above which Pd atoms become stable on the surface of NP_201_; we plot this value in Figure 7 for Wulff-type particles of increasing size (circles in Figure 7) ranging from NP_201_ to NP_12934_ (*D* = 7.8 nm). Below this line the whole Pd fraction is expected to reside in the core of NPs confined by purely Au shell ($\theta_{terrace}^{Pd}=0$).

From the data in Figure 7 one can expect appearance of Pd1 monomers on the surface of NP_586_ for ca. 1:2 Pd:Au ratio. Indeed, applying the TOP approach to Pd_199_Au_387_ and Pd_219_Au_367_ compositions of NP_586_ resulted in the lowest-energy homotops with $\theta_{terrace}^{Pd}$ of 0.02 (6 Pd1 monomers) and 0.08 (12 Pd1), respectively. The corresponding Pd fractions in the core, $\rho_{core}^{Pd}$, are 0.62 and 0.66. The topological energy descriptors of Pd_56_Au_145_ composition were used here and the obtained $\rho_{core}^{Pd}$ and $\theta_{terrace}^{Pd}$ values are quite consistent with the trend shown in Figure 1.

**Figure 8:**
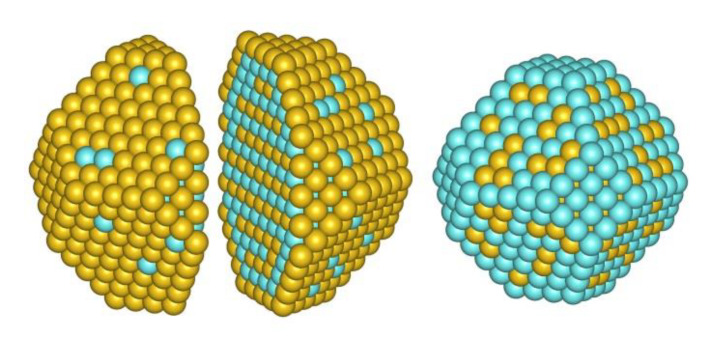
The structure of Pd_511_Au_778_ (NP_1289_) found after 10 million MC steps to minimize the topological energy, Equation (1), using the descriptors for the NP_201_ composition Pd_56_Au_145_: the outer shell consisting of 482 atoms (**left** and **center**), cross section (**center**) and the subsurface shell (**right**).

Furthermore, employing the same approach to Pd_511_Au_778_ composition of NP_1289_ (Figure 8) one obtains $\theta_{terrace}^{Pd}$ = 0.11 and $\rho_{core}^{Pd}$ = 0.60, in agreement with the estimations derived from Figure 7 and Figure 1. The Pd_511_Au_778_ homotop displayed in Figure 8 exhibits the atomic ordering similar to that of NP_201_ at similar $\rho_{core}^{Pd}$ and $\theta_{terrace}^{Pd}$ values: Pd species are scattered in the outer shell as monomers (27 Pd1 in total) and dimers (3 Pd2), while the subsurface shell is enriched by Pd. Noteworthy, edges of the subsurface shell show preference for hosting Pd atoms, while Au atoms are located on terraces of this shell. The average number of Pd-Au bonds per Pd atom in Pd_511_Au_778_ is 7.4, slightly lower than the value 8.3 found for Pd_56_Au_145_.

Figure 7 also shows the region where the bulk-like PdAu_3_ ordering is expected: the dashed line corresponds to $\rho_{core}^{Pd}$ = 0.25 (diamonds in Figure 7 correspond to the nuclearities of Wulff-type NPs). Below this line Pd fraction is distributed in the form of Pd monomers in the core of NPs. It is clearly seen in Figure 7 that for 5–8 nm large particles the fraction of surface atoms (Au shell) is small compared to the core. Therefore, the total Pd/Au ratio required for the PdAu_3_ phase L1_2_ to be formed in the core is only slightly lower than 0.25. Indeed, L1_2_ phase was reported for NPs larger than 5 nm [46]. However, lower Pd/Au ratios appear to be optimal for the core ordering PdAu_3_ in smaller NPs, e.g., ~0.15 and ~0.2 for 2.5 and 4 nm large NPs, respectively.

The activity and selectivity of bimetallic catalysts are often discussed using the concept of the ensemble (geometric) and ligand (electronic) effects [13,48,49,50,51]. These effects originate from the formation of contiguous palladium domains, when diluting Pd with Au, and charge transfer between Au and Pd or orbital re-hybridization of metal atoms involved in heteronuclear metal-metal bonds. Most studies have been focused on the ensemble effect. The catalytic performance of PdAu nanoalloys strongly correlates with the Pd/Au ratio. The current work provides the relationship between the Pd/Au ratio in core of bimetallic particles, particles size, and surface structure of the Pd sites. Such data could be used as a basis for synthesis of Pd single-site catalysts for application in a number of catalytic reactions.

## 4. Conclusions

Using DFT approach and DFT-parameterized topological energy expressions we studied the distribution of metal components in bimetallic PdAu nanoparticles. One of the main findings is that already at very low temperature single-atom Pd1 centers become stable in the outer surface shell of nanoparticles formed by Au atoms at the concentration of Pd in the particle core exceeding ca. 60%. The present modeling approach can be extended to other nanoalloys in order to establish their compositions at which single-atom surface centers are stable. Such data will pave the way to approaching elusive aim of the knowledge-driven designing very selective catalysts exposing active metal sites of just one type.

The general mechanism of Pd and Au ordering in PdAu nanoparticles can be rationalized by interplay of two effects: (i) the miscibility of Pd and Au manifesting itself in maximizing the number of Pd-Au bonds in the minimum-energy atomic orderings and (ii) the stabilization of purely Au atomic outer shell of the nanoparticles, when Pd concentration in their inner region is lower than the threshold value. Concentration of the surface Pd1 centers is a function of Pd concentration in the particle core.

Analysis of structural changes with the increase of Pd fraction shows various ordering types in the cores of model nanoparticles. At very low Pd concentrations the whole Pd fraction is distributed in the core as Pd1 monomers coordinated by 12 Au atoms. When Pd concentration in the core is increased to ca. 0.25, ordered bulk-like PdAu_3_ structures confined by the gold outer shell are formed. Interestingly, for small particles such as NP_201_ the emergence of either L1_2_ (Pd_20_Au_181_) or D0_22_ (Pd_22_Au_179_) orderings is determined by a tiny variation of the Pd:Au ratio. Further increase of Pd fraction leads to more dense Pd distribution in the core with formation of Pd-Pd bonds. Non-bulk orderings in the core accompany formation of stable surface Pd1 monomers: The subsurface shell is enriched by Pd with the edges of this shell fully occupied by Pd atoms, while Au atoms are located in (111) terraces of the subsurface shell. Thus, the occupation order of terrace-edge positions in the subsurface shell of a PdAu nanoparticle is inversed for Pd and Au with respect to the outer shell.

## Figures and Tables

**Table 1:** Energy descriptors in the topological expression Equation (1) parameterized for various Pd_x_Au_201−x_ nanoparticle (NP) compositions along with accompanying data *^a^.*

Parameter	Pd_56_Au_145_ *^b^*	Pd_79_Au_122_	Pd_101_Au_100_
$E_{0}$, eV	−668.8974	−707.8834	−745.6012
$\varepsilon_{CORNER}^{Au}$, eV	−0.3702	−0.3854	−0.4574
$\varepsilon_{EDGE}^{Au}$, eV	−0.3364	−0.3452	−0.3991
$\varepsilon_{TERRACE}^{Au}$, eV	−0.1922	−0.2500	−0.2872
$\varepsilon_{BOND}^{Au-Pd}$, eV	−0.0263	−0.0248	−0.0225
$\varepsilon_{TERRACE}^{Au}$/$\varepsilon_{BOND}^{Au-Pd}$	7.3	10.1	12.8
*N*	44	28	32
Δ*E*, eV	0.0000	0.0320	0.0000
δ, eV	0.0934	0.2225	0.2974

*^a^ N* is the number of DFT calculations performed to define Equation (1). The accuracy Δ*E* is the energy difference (within *E*_TOP_ data) between the lowest-energy homotop obtained in the DFT calculations and the lowest-energy homotop obtained in the TOP optimization. The precision δ is twice the residual standard deviation between *E*_TOP_ and *E*_DFT_ energies for arbitrarily chosen, 10-test low-energy homotops different from the homotops used to construct Equation (1). According to this definition relative TOP energies for each pair of homotops are within *δ* of the corresponding DFT energy differences with the probability >95% [20]. *^b^* Descriptors for Pd_56_Au_145_ are the same as in the previous publication [14].

**Table 2:** Numbers of Pd atoms in the core, $n_{core}^{Pd}$, and surface (111) terrace, $n_{terrace}^{Pd}$, sites of NP_201_ at various compositions Pd_201−x_Au_x_ and the corresponding Pd concentrations, $\rho_{core}^{Pd}$ and $\theta_{terrace}^{Pd}$.

	$n_{core}^{Pd}$	$\rho_{core}^{Pd}$	$n_{terrace}^{Pd}$	$\theta_{terrace}^{Pd}$
Pd_19_Au_182_	19	0.24	0	0
Pd_44_Au_157_	44	0.56	0	0
Pd_51_Au_150_	48	0.60	3	0.05
Pd_56_Au_145_	50	0.63	6	0.11
Pd_62_Au_139_	56	0.71	6	0.11
Pd_67_Au_134_	60	0.76	7	0.125
Pd_73_Au_128_	63	0.80	10	0.18
Pd_79_Au_122_	63	0.80	16	0.29
Pd_101_Au_100_	76	0.96	24 *^a^*	0.41

*^a^* Including one atom on (001) terrace.

## Data Availability

The data presented in this study are available in Supplementary Materials.

## Supplementary Materials

The following data are available online at https://www.mdpi.com/2079-4991/11/1/122/s1, Figure S1: Topological energy descriptors as function of Pd:Au ratio in the compositions PdmAu201−m (m = 56, 79, 101) of model NP201; Table S1: Topological energy descriptors for compositions PdmAu201−m (m= 51, 62, 67, 73) of NP201; Table S2: Energetic and topological characteristics of low-lying homotops of PdmAu201−m compositions with m = 51, 56, 62, 67, 73, 79; Table S3: Energetic and topological characteristics of low-lying homotops of Pd101Au100 composition.
