# Supplementary material for: Pd Single-Atom Sites on the Surface of PdAu Nanoparticles: A DFT-Based Topological Search for Suitable Compositions
Source: Nanomaterials (Basel). 2021 Jan 7;11(1):122. doi: 10.3390/nano11010122 (PMC7828025; doi:10.3390/nano11010122)
Supplement: Supplementary file 1 [file nanomaterials-11-00122-s001.pdf]

Supplementary Materials

Pd Single-Atom Sites on the Surface of PdAu Nanoparticles: A DFT-Based Topological Search for Suitable Compositions

Mikhail Mamatkulov[1], Ilya V. Yudanov[1,*], Andrey V. Bukhtiyarov[1] and Konstantin M. Neyman[2,3,*]

[1] Boreskov Institute of Catalysis of the Siberian Branch of the Russian Academy of Sciences (BIC SB RAS), 630090 Novosibirsk, Russia; mikhail@catalysis.ru (M.M); yudanov@catalysis.ru (I.V.Y.); avb@catalysis.ru (A.V.B.)

[2] Departament de Ciència de Materials i Química Física and Institut de Quimica Teòrica i Computacional, Universitat de Barcelona, c/Martí i Franquès 1, 08028 Barcelona, Spain; konstantin.neyman@ub.edu

[3] ICREA (Institució Catalana de Recerca i Estudis Avançats), Pg. Lluís Companys 23, 08010 Barcelona, Spain; konstantin.neyman@icrea.cat

* Correspondence: yudanov@catalysis.ru (I.V.Y.); konstantin.neyman@icrea.cat (K.M.N.)

Content of Supplementary Materials:

Figure S1. Topological energy descriptors as function of Pd:Au ratio in the compositions Pd_mAu_{201-m} (m = 56, 79, 101) of model NP_{201}.

Table S1. Topological energy descriptors for the compositions Pd_mAu_{201-m} (m= 51, 62, 67, 73) of model NP_{201}.

Table S2. Energetic and topological characteristics of low-lying homotops of Pd_mAu_{201-m} compositions with m = 51, 56, 62, 67, 73, 79.

Table S3. Energetic and topological characteristics of low-lying homotops of $Pd_{101}Au_{100}$ composition.

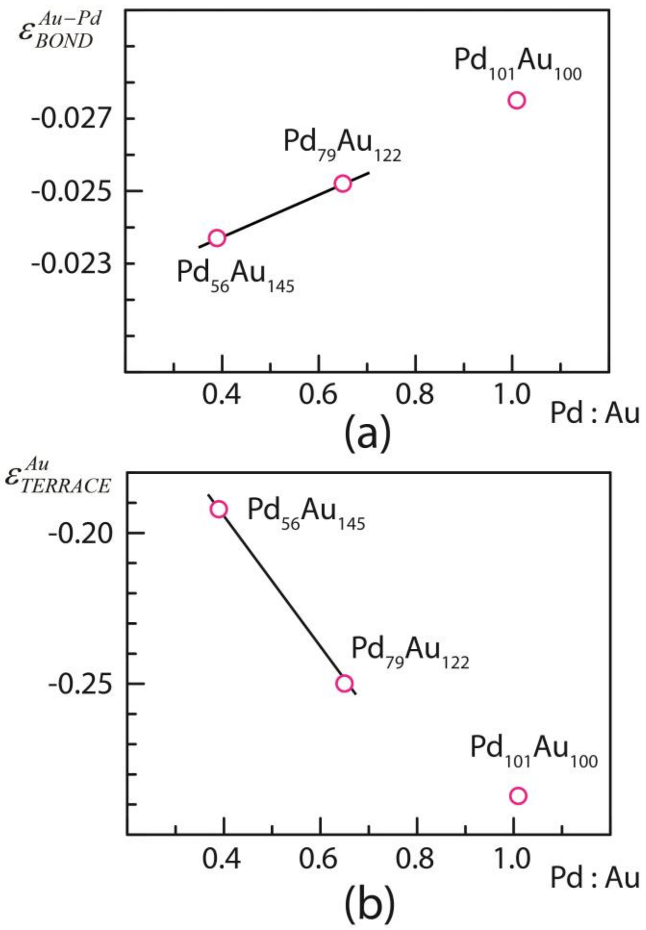

Figure S1. Topological energy descriptors $\varepsilon_{BOND}^{Au-Pd}$ (**a**) and $\varepsilon_{TERRACE}^{Au}$ (**b**) as function of Pd:Au ratio in the compositions Pd_mAu_{201-m} (m = 56, 79, 101) of model NP_{201}. Full list of the descriptors is presented in Table 1. The linear approximations (shown by solid lines) were used to obtain the topological energy descriptors for compositions between $Pd_{56}Au_{145}$ and $Pd_{79}Au_{122}$ ones (see Table S1).

Table S1. Topological energy descriptors for the compositions Pd_mAu_{201-m} (m= 51, 62, 67, 73) based on linear approximations (the examples of $\varepsilon_{BOND}^{Au-Pd}$ and $\varepsilon_{TERRACE}^{Au}$ are shown in Fig. S1) of the descriptors optimized for $Pd_{56}Au_{145}$ and $Pd_{79}Au_{122}$.

	$Pd_{51}Au_{150}$	$Pd_{62}Au_{139}$	$Pd_{67}Au_{134}$	$Pd_{73}Au_{128}$
E_0	-662.001	-677.821	-685.870	-696.359
$\varepsilon_{BOND}^{Au-Pd}$	-0.0266	-0.0260	-0.0257	-0.0252
$\varepsilon_{TERRACE}^{Au}$	-0.1820	-0.2055	-0.2174	-0.2330
ε_{EDGE}^{Au}	-0.3349	-0.3384	-0.3403	-0.3426
$\varepsilon_{CORNER}^{Au}$	-0.3675	-0.3737	-0.3768	-0.3809
$\varepsilon_{TERRACE}^{Au} / \varepsilon_{BOND}^{Au-Pd}$	6.84	7.91	8.48	9.24

Table S2. Energetic and topological characteristics of low-lying homotops of the compositions Pd_mAu_{201-m} (m = 51, 56, 62, 67, 73, 79). The total energies E_{DFT} and E_{TOP} are calculated by TOP and DFT approaches, respectively. The relative energies ΔE_{DFT} and ΔE_{TOP} are calculated with respect to the homotop with the lowest E_{DFT}. In the listed homotops the sites on edges, corners and (001) facets are occupied by Au atoms. The surface Pd atoms, $N_{TERRACE}^{Pd}$ (if any), are located on (111) facets.

Homotop number in NP_{201}	E_{DFT}	ΔE_{DFT}	E_{TOP}	ΔE_{TOP}	N_{BOND}^{Au-Pd}	N_{CORE}^{Au}	$N_{TERRACE}^{Au}$	$N_{TERRACE}^{Pd}$
**$Pd_{51}Au_{150}$**								
1	-703.436	0.000	-705.186	0.000	435	31	53	3
2	-703.293	0.143	-705.191	-0.004	442	32	52	4
3	-703.270	0.166	-705.178	0.008	421	29	55	1
4	-703.259	0.177	-705.195	-0.008	449	33	51	5
5	-703.248	0.188	-705.174	0.013	414	28	56	0
6	-703.232	0.204	-705.125	0.062	419	29	55	1
7	-703.229	0.207	-705.121	0.066	412	28	56	0
8	-703.227	0.209	-705.186	0.000	435	31	53	3
9	-703.220	0.216	-705.199	-0.013	456	34	50	6
10	-703.202	0.234	-705.027	0.160	429	31	53	3
11	-703.191	0.245	-705.203	-0.017	463	35	49	7
12	-703.173	0.263	-705.182	0.004	428	30	54	2
13	-703.158	0.278	-705.133	0.053	433	31	53	3
14	-703.125	0.311	-705.018	0.168	415	29	55	1
15	-703.119	0.317	-705.101	0.085	466	36	48	8
16	-703.114	0.322	-705.076	0.111	424	30	54	2
17	-703.104	0.332	-705.072	0.115	417	29	55	1
18	-703.091	0.345	-704.908	0.279	404	28	56	0
19	-703.070	0.366	-704.969	0.217	420	30	54	2
20	-703.063	0.373	-704.859	0.328	409	29	55	1
21	-702.983	0.453	-704.810	0.377	414	30	54	2
22	-702.975	0.461	-704.916	0.270	418	30	54	2
23	-702.914	0.522	-704.646	0.540	401	29	55	1
24	-702.911	0.525	-704.757	0.430	412	30	54	2
25	-702.866	0.570	-704.703	0.483	410	30	54	2
26	-702.828	0.608	-704.892	0.294	465	37	47	9
27	-702.576	0.860	-704.631	0.556	462	38	46	10

Table S2. Continued.

Homotop number in NP_{201}	E_{DFT}	ΔE_{DFT}	E_{TOP}	ΔE_{TOP}	N_{BOND}^{Au-Pd}	N_{CORE}^{Au}	$N_{TERRACE}^{Au}$	$N_{TERRACE}^{Pd}$
**$Pd_{56}Au_{145}$**								
1	-712.881	0.000	-712.863	0.000	464	29	50	6
2	-712.862	0.019	-712.810	0.053	462	29	50	6
3	-712.856	0.025	-712.847	0.016	478	31	48	8
4	-712.853	0.028	-712.819	0.044	455	28	51	5
5	-712.834	0.047	-712.802	0.061	469	30	49	7
6	-712.822	0.059	-712.794	0.069	476	31	48	8
7	-712.804	0.077	-712.766	0.097	453	28	51	5
8	-712.802	0.079	-712.758	0.105	460	29	50	6
9	-712.801	0.080	-712.855	0.008	471	30	49	7

10	-712.798	0.083	-712.750	0.113	467	30	49	7
11	-712.788	0.093	-712.827	0.036	448	27	52	4
12	-712.783	0.098	-712.899	-0.036	480	31	48	8
13	-712.777	0.104	-712.705	0.158	458	29	50	6
14	-712.768	0.113	-712.713	0.150	451	28	51	5
15	-712.740	0.141	-712.722	0.142	444	27	52	4
16	-712.730	0.151	-712.705	0.158	458	29	50	6
17	-712.718	0.163	-712.697	0.166	465	30	49	7
18	-712.685	0.196	-712.653	0.210	456	29	50	6
19	-712.654	0.227	-712.616	0.247	440	27	52	4
20	-712.643	0.238	-712.608	0.255	447	28	51	5
21	-712.631	0.250	-712.661	0.202	449	28	51	5
22	-712.591	0.290	-712.628	0.235	477	32	47	9
23	-712.577	0.304	-712.754	0.109	416	23	56	0
24	-712.548	0.333	-712.596	0.267	410	23	56	0
25	-712.543	0.338	-712.527	0.336	422	25	54	2
26	-712.505	0.376	-712.484	0.379	477	32	48	8
27	-712.502	0.379	-712.422	0.441	418	25	54	2
28	-712.494	0.387	-712.390	0.473	446	29	50	6
29	-712.493	0.388	-712.701	0.162	414	23	56	0
30	-712.477	0.404	-712.345	0.518	437	28	51	5
31	-712.471	0.410	-712.467	0.396	427	26	53	3
32	-712.411	0.470	-712.414	0.449	425	26	53	3
33	-712.390	0.491	-712.329	0.534	451	30	49	7
34	-712.380	0.501	-712.353	0.510	430	27	52	4
35	-712.339	0.542	-712.276	0.587	449	30	49	7
36	-712.193	0.688	-712.119	0.745	443	30	49	7
37	-712.146	0.735	-712.110	0.753	450	31	48	8
38	-712.012	0.869	-712.005	0.858	446	31	48	8

Table S2. Continued.

Homotop number in NP_{201}	E_{DFT}	ΔE_{DFT}	E_{TOP}	ΔE_{TOP}	N_{BOND}^{Au-Pd}	N_{CORE}^{Au}	$N_{TERRACE}^{Au}$	$N_{TERRACE}^{Pd}$
**$Pd_{62}Au_{139}$**								
1	-723.795	0.00	-722.3362	0.000	456	23	50	6
2	-723.772	0.02	-722.2137	0.122	475	26	47	9
3	-723.755	0.04	-722.3412	-0.005	472	25	48	8
4	-723.732	0.06	-722.3077	0.029	447	22	51	5
5	-723.718	0.08	-722.3312	0.005	440	21	52	4
6	-723.712	0.08	-722.2372	0.099	468	25	48	8
7	-723.708	0.09	-722.3127	0.024	463	24	49	7
8	-723.692	0.10	-722.1852	0.151	466	25	48	8
9	-723.691	0.10	-722.2892	0.047	470	25	48	8
10	-723.674	0.12	-722.3547	-0.019	433	20	53	3
11	-723.657	0.14	-722.1332	0.203	464	25	48	8
12	-723.657	0.14	-722.2087	0.128	459	24	49	7
13	-723.655	0.14	-722.0812	0.255	462	25	48	8
14	-723.654	0.14	-722.2322	0.104	452	23	50	6
15	-723.638	0.16	-722.1382	0.198	480	27	46	10
16	-723.637	0.16	-722.3782	-0.042	426	19	54	2
17	-723.629	0.17	-722.3497	-0.014	417	18	55	1

18	-723.610	0.19	-722.1567	0.180	457	24	49	7
19	-723.608	0.19	-722.0577	0.279	469	26	47	9
20	-723.600	0.19	-722.1802	0.156	450	23	50	6
21	-723.600	0.19	-722.0627	0.274	485	28	45	11
22	-723.567	0.23	-722.0392	0.297	492	29	44	12
23	-723.404	0.39	-721.9117	0.424	495	30	43	13
24	-723.338	0.46	-721.6567	0.679	501	32	41	15
25	-723.330	0.47	-721.7842	0.552	498	31	42	14
26	-723.127	0.67	-721.5292	0.807	504	33	40	16
27	-722.775	1.02	-721.4017	0.934	507	34	39	17
28	-722.706	1.09	-721.2742	1.062	510	35	38	18

Table S2. Continued.

Homotop number in NP_{201}	E_{DFT}	ΔE_{DFT}	E_{TOP}	ΔE_{TOP}	N^{Au-Pd}_{BOND}	N^{Au}_{CORE}	$N^{Au}_{TERRACE}$	$N^{Pd}_{TERRACE}$
**$Pd_{67}Au_{134}$**								
1	-732.4618	0.000	-730.712	0.000	451	19	49	7
2	-732.4613	0.000	-730.674	0.038	458	20	48	8
3	-732.4471	0.015	-730.698	0.014	442	18	50	6
4	-732.4289	0.033	-730.581	0.131	429	17	51	5
5	-732.4197	0.042	-730.660	0.051	449	19	49	7
6	-732.4122	0.050	-730.571	0.140	454	20	48	8
7	-732.4042	0.058	-730.609	0.103	447	19	49	7
8	-732.3997	0.062	-730.534	0.178	461	21	47	9
9	-732.3970	0.065	-730.684	0.028	433	17	51	5
10	-732.3776	0.084	-730.567	0.144	420	16	52	4
11	-732.3705	0.091	-730.520	0.192	452	20	48	8
12	-732.3318	0.130	-730.445	0.267	466	22	46	10
13	-732.3195	0.142	-730.459	0.253	475	23	45	11
14	-732.3055	0.156	-730.502	0.210	409	15	53	3
15	-732.2990	0.163	-730.506	0.206	443	19	49	7
16	-732.2957	0.166	-730.544	0.168	436	18	50	6
17	-732.2918	0.170	-730.421	0.290	482	24	44	12
18	-732.2855	0.176	-730.295	0.417	494	26	42	14
19	-732.2640	0.198	-730.614	0.097	388	12	56	0
20	-732.2590	0.203	-730.384	0.328	489	25	43	13
21	-732.2386	0.223	-730.366	0.346	446	20	48	8
22	-732.2164	0.245	-730.417	0.294	448	20	48	8
23	-732.2005	0.261	-730.206	0.506	499	27	41	15
24	-732.1938	0.268	-730.389	0.322	430	18	50	6
25	-732.1837	0.278	-730.314	0.397	444	20	48	8
26	-732.0744	0.387	-730.166	0.546	379	13	55	1
27	-732.0591	0.403	-730.028	0.683	509	29	39	17
28	-731.9624	0.499	-730.025	0.686	382	14	54	2
29	-731.9514	0.510	-729.888	0.824	512	30	38	18
30	-731.8487	0.613	-729.748	0.964	515	31	37	19
31	-731.7790	0.683	-729.860	0.851	494	28	40	16

Table S2. Continued.

Homotop number in NP_{201}	E_{DFT}	ΔE_{DFT}	E_{TOP}	ΔE_{TOP}	N_{BOND}^{Au-Pd}	N_{CORE}^{Au}	$N_{TERRACE}^{Au}$	$N_{TERRACE}^{Pd}$
**$Pd_{73}Au_{128}$**								
1	-742.829	0.000	-708.149	0.000	450	16	46	10
2	-742.695	0.134	-708.092	0.057	457	17	45	11
3	-742.680	0.149	-708.117	0.032	421	13	49	7
4	-742.674	0.155	-708.111	0.038	430	14	48	8
5	-742.670	0.159	-708.104	0.044	439	15	47	9
6	-742.666	0.163	-708.042	0.107	455	17	45	11
7	-742.577	0.252	-708.035	0.113	464	18	44	12
8	-742.565	0.264	-707.991	0.157	453	17	45	11
9	-742.544	0.285	-708.048	0.101	446	16	46	10
10	-742.543	0.286	-708.073	0.076	410	12	50	6
11	-742.530	0.299	-708.079	0.070	401	11	51	5
12	-742.512	0.317	-708.085	0.064	392	10	52	4
13	-742.474	0.355	-707.809	0.340	492	22	40	16
14	-742.460	0.369	-707.771	0.378	472	20	42	14
15	-742.435	0.394	-708.004	0.145	435	15	47	9
16	-742.409	0.420	-707.866	0.283	485	21	41	15
17	-742.407	0.422	-707.997	0.151	444	16	46	10
18	-742.400	0.429	-707.979	0.170	471	19	43	13
19	-742.388	0.441	-707.947	0.202	442	16	46	10
20	-742.387	0.442	-707.890	0.258	449	17	45	11
21	-742.367	0.462	-707.752	0.396	499	23	39	17
22	-742.324	0.505	-707.834	0.315	456	18	44	12
23	-742.320	0.509	-708.041	0.108	381	9	53	3
24	-742.289	0.540	-708.047	0.102	372	8	54	2
25	-742.282	0.547	-708.053	0.095	363	7	55	1
26	-742.227	0.602	-707.645	0.503	504	24	38	18
27	-742.218	0.611	-707.721	0.428	470	20	42	14
28	-742.193	0.636	-707.488	0.661	507	25	37	19
29	-742.181	0.648	-707.664	0.485	477	21	41	15

Table S2. Continued.

Homotop number in NP_{201}	E_{DFT}	ΔE_{DFT}	E_{TOP}	ΔE_{TOP}	N_{BOND}^{Au-Pd}	N_{CORE}^{Au}	$N_{TERRACE}^{Au}$	$N_{TERRACE}^{Pd}$
**$Pd_{79}Au_{122}$**								
1	-752.901	0.000	-752.661	0.000	468	16	40	16
2	-752.890	0.011	-752.661	0.000	468	16	40	16
3	-752.859	0.042	-752.661	0.000	468	16	40	16
4	-752.779	0.122	-752.692	-0.031	439	13	43	13
5	-752.742	0.159	-752.584	0.077	475	17	39	17
6	-752.690	0.211	-752.611	0.050	466	16	40	16
7	-752.646	0.255	-752.535	0.126	473	17	39	17
8	-752.585	0.316	-752.620	0.041	426	12	44	12
9	-752.579	0.322	-752.724	-0.063	410	10	46	10

10	-752.544	0.357	-752.431	0.230	489	19	37	19
11	-752.542	0.359	-752.490	0.171	451	15	41	15
12	-752.530	0.371	-752.539	0.122	453	15	41	15
13	-752.525	0.376	-752.589	0.072	455	15	41	15
14	-752.522	0.379	-752.697	-0.036	419	11	45	11
15	-752.510	0.391	-752.440	0.221	449	15	41	15
16	-752.501	0.400	-752.643	0.018	437	13	43	13
17	-752.498	0.403	-752.616	0.045	446	14	42	14
18	-752.497	0.404	-752.701	-0.040	399	9	47	9
19	-752.484	0.417	-752.647	0.014	417	11	45	11
20	-752.483	0.418	-752.566	0.095	444	14	42	14
21	-752.483	0.418	-752.755	-0.094	381	7	49	7
22	-752.475	0.426	-752.593	0.068	435	13	43	13
23	-752.467	0.434	-752.728	-0.067	390	8	48	8
24	-752.457	0.444	-752.728	-0.067	390	8	48	8
25	-752.434	0.467	-752.305	0.356	494	20	36	20
26	-752.427	0.474	-752.503	0.158	391	9	47	9
27	-752.396	0.505	-752.337	0.324	465	17	39	17
28	-752.391	0.510	-752.214	0.447	363	7	50	6
29	-752.358	0.543	-752.359	0.302	476	18	38	18
30	-752.357	0.544	-752.314	0.347	454	16	40	16
31	-752.325	0.576	-752.111	0.550	487	19	39	17
32	-752.316	0.585	-752.260	0.401	472	18	38	18
33	-752.252	0.649	-752.399	0.262	431	13	44	12
34	-752.227	0.674	-752.188	0.473	459	17	39	17
35	-752.185	0.716	-752.138	0.523	457	17	39	17
36	-752.107	0.794	-751.903	0.758	276	0	56	0

Table S3. Energetic and topological characteristics of low-lying homotops of $Pd_{101}Au_{100}$ composition.

	E_{DFT}	ΔE_{DFT}	E_{TOP}	ΔE_{TOP}	N_{BOND}^{Au-Pd}	N_{CORE}^{Au}	N_{EDGE}^{Au}	N_{CORNER}^{Au}	N_{001}^{Pd}	N_{111}^{Pd}
1	-790.314	0.000	-790.216	0.000	384	3	36	24	1	24
2	-790.295	0.018	-790.132	0.084	342	0	36	24	0	22
3	-790.286	0.027	-790.132	0.084	342	0	36	24	0	22
4	-790.245	0.068	-790.233	-0.017	372	2	36	24	0	24
5	-790.228	0.086	-790.087	0.129	340	0	36	24	0	22
6	-790.205	0.108	-790.171	0.045	382	3	36	24	1	24
7	-790.198	0.116	-790.148	0.068	381	3	36	24	0	25
8	-790.152	0.161	-789.997	0.219	336	0	36	24	0	22
9	-790.143	0.170	-790.042	0.174	338	0	36	24	0	22
10	-790.112	0.201	-790.193	0.023	383	3	36	24	0	25
11	-790.104	0.209	-790.076	0.140	365	2	36	24	1	23
12	-790.102	0.212	-790.065	0.151	339	0	36	24	1	21
13	-790.101	0.212	-789.997	0.219	336	0	36	24	0	22
14	-790.044	0.270	-790.008	0.208	362	2	36	24	0	24
15	-790.038	0.275	-790.131	0.085	393	4	36	24	1	25
16	-790.032	0.281	-789.905	0.311	365	2	36	23	0	23
17	-790.024	0.289	-789.975	0.241	335	0	36	24	1	21
18	-790.020	0.294	-789.855	0.361	350	1	36	23	0	22
19	-789.998	0.316	-790.042	0.174	394	4	35	24	0	25
20	-789.994	0.320	-790.199	0.017	396	4	36	24	2	24
21	-789.992	0.322	-790.081	0.135	378	3	36	24	1	24
22	-789.968	0.346	-790.199	0.017	396	4	36	24	2	24
23	-789.934	0.380	-789.913	0.303	345	1	36	24	0	23
24	-789.926	0.388	-790.031	0.185	363	2	36	24	1	23
25	-789.917	0.397	-789.953	0.263	339	0	35	24	1	20
26	-789.900	0.414	-790.154	0.062	394	4	36	24	2	24
27	-789.863	0.450	-789.930	0.286	333	0	36	24	1	21
28	-789.858	0.456	-790.069	0.147	403	5	36	24	0	27
29	-789.841	0.473	-789.839	0.377	380	4	36	24	0	26
30	-789.836	0.478	-790.013	0.203	375	3	36	24	0	25
31	-789.832	0.482	-789.952	0.264	334	0	36	24	2	20
32	-789.824	0.490	-789.980	0.236	348	1	36	24	1	22
33	-789.819	0.494	-789.980	0.236	348	1	36	24	1	22
34	-789.796	0.517	-790.029	0.187	414	6	36	24	0	28
35	-789.796	0.517	-789.890	0.326	344	1	36	24	1	22
36	-789.778	0.536	-790.013	0.203	375	3	36	24	2	23
